# Synthetic microbial communities: emerging live biotherapeutics for targeted gut microbiome modulation

**DOI:** 10.1080/19490976.2026.2719056

**Published:** 2026-08-15

**Authors:** Ru Zhang, Hongye Li, Chunfeng Wang, Guilian Yang

**Affiliations:** a College of Veterinary Medicine, Jilin Agricultural University, Changchun, People's Republic of China; b Jilin Provincial Engineering Research Center of Animal Probiotics, Jilin Provincial Key Laboratory of Animal Microecology and Healthy Breeding, Jilin Agricultural University, Changchun, People's Republic of China; c Engineering Research Center of Microecological Vaccines (Drugs) for Major Animal Diseases, Ministry of Education, Jilin Agricultural University, Changchun, People's Republic of China; d Institute of Special Animal and Plant Sciences of CAAS, Changchun, People's Republic of China

**Keywords:** Synthetic microbial communities, intestinal diseases, live biotherapeutic products, rational design, microbiota-based therapies

## Abstract

Gut microbiome dysbiosis causes various intestinal diseases. However, an undefined composition and potential biosafety risks limit the applicability of traditional fecal microbiota transplantation (FMT). Synthetic microbial communities (SynComs), which are compositionally defined and rationally designed emerging live biotherapeutics, offer a novel alternative to FMT. This review establishes strict boundaries between SynComs and traditional donor-derived preparations, comparatively evaluating “top-down” and “bottom-up” construction strategies. We explored the mechanisms underlying the SynComs-mediated synergistic restoration of intestinal homeostasis via direct targeted antagonism and modulation of the host immune network. Moreover, we systematically evaluated the current research landscape of SynComs in Clostridioides difficile infection, inflammatory bowel disease, and colorectal cancer. This review examines fundamental challenges, including host colonization resistance, chemistry, manufacturing, and control barriers, biosafety risks, and microbiokinetic regulatory frameworks, thereby addressing the translational gap. Our analysis of current literature provides a theoretical basis for the clinical translation of SynComs as emerging live biotherapeutics.

## 1. Introduction

The gut microbiota modulates several host physiological processes. It maintains the dynamic balance of mucosal barrier integrity, metabolic homeostasis, and immune axes. Thus, it is recognized as a critical metabolic and immunoregulatory hub for health maintenance.^1^ However, traditional microecological intervention strategies have considerable limitations in addressing complex microbial dysbiosis. Commercial probiotics often lack robust colonization capacity tailored to specific disease contexts, whereas the “black-box” nature of fecal microbiota transplantation (FMT) introduces risks such as undefined compositional profiles, inter-batch variability in therapeutic efficacy, and horizontal gene transfer of antimicrobial resistance determinants.^2^ To address compositional ambiguity and donor dependency, the field of microecological therapeutics is accelerating its transition from the paradigm of “empirical whole-community transfer” toward one of “rational ecological reconstruction.”

Synthetic microbial communities (SynComs) have recently emerged as advanced modalities for live biotherapeutic products (LBPs). SynComs do not comprise a random assemblage of strains; rather they are rationally assembled “white-box” systems engineered in accordance with synthetic biology principles and constructed from modular strains with well-defined genetic backgrounds and complementary ecological niches.^3^ ^,^ ^4^ Contrary to traditional therapeutic approaches, engineered SynComs are designed not only to remodel host antimicrobial barriers and immune tolerance through multidimensional ecological networks but also to incorporate environmentally responsive genetic elements that enable dynamic sensing of pathological microenvironmental signals. This design concept makes SynComs a promising research platform for achieving precise modulation and context-responsive intervention in the gut microecology.^5^

Mechanistically, SynComs exert their therapeutic effects through two synergistic pathways: direct antagonism and indirect modulation of host immunity. Direct antagonism acts through resource competition (e.g., deprivation of essential nutrients), physical niche occupation, and targeted release of antimicrobial metabolites–including bacteriocins, short-chain fatty acids (SCFAs), and secondary bile acids–to directly suppress pathogen colonization and proliferation.^6^ In turn, indirect modulation of host immunity reshapes intestinal homeostasis via metabolic signaling molecules such as butyrate-mediated inhibition of histone deacetylases (HDACs) and regulation of the farnesoid X receptor (FXR) and G protein-coupled receptor 5 (TGR5) by secondary bile acids, thereby inducing regulatory T (Treg) cell differentiation and maintaining Treg/Th17 immune equilibrium.^7^ ^,^ ^8^ More importantly, these two mechanisms are not merely additive in function; rather, they can be harnessed through engineering design to achieve spatiotemporal coordination and context-dependent coupling.

This review integrates the current knowledge on design principles, mechanistic underpinnings, and translational evidence for SynComs, with a specific emphasis on their distinct application profiles across *Clostridioides difficile* infection (CDI), inflammatory bowel disease (IBD), and colorectal cancer (CRC). Beyond mechanistic recapitulation, we critically examined the major challenges that prevent clinical application, including colonization resistance imposed by resident microbes, challenges in maintaining defined compositional stoichiometry during scale-up manufacturing, biosafety implications of horizontal gene transfer, and the absence of validated pharmacokinetic/pharmacodynamic (PK/PD) paradigms for live biotherapeutics. Collectively, our objective was to provide a conceptually rigorous and practical framework for guiding the rational development of SynComs as a new class of microbiome-targeted therapeutics.

## 2. SynComs: concept, design principles, and design strategies

### 2.1. Concept

SynComs are stable consortia assembled according to the principles of synthetic biology through the rational selection of microorganisms, including prokaryotic, eukaryotic, and engineered strains with well-characterized genetic backgrounds. They are designed to achieve compositionally defined, functionally pre-specified, and ecologically robust ensembles.^3^ The emergence of SynComs indicates a conceptual transition in microecological interventions. Specifically, it demonstrates a shift from the traditional paradigm of “empirically driven whole-community transfer” toward “rational design and systems-level reconstitution.”

Within the spectrum of LBPs, products should be rigorously distinguished based on their technological routes and compositional boundaries. Traditional probiotics and single-strain LBPs typically have narrow functional spectra.^9^ Although FMT and its derivative spore-based products (e.g., SER-109) have demonstrated clinical efficacy for specific indications, they originate from non-isolated donor feces. Thus, they remain essentially complex consortia with undefined compositions and cannot fully circumvent donor dependency or the potential risk of pathogen transmission.^10^ Donor-independent compositionally defined microbiota therapeutics (e.g., VE303 and MET-2) have been sequentially developed to address these limitations.^11^ ^,^ ^12^ Building on this foundation, SynComs—designed with niche complementarity as a core principle and further engineered as microbial consortia incorporating genetic circuits—represent the next evolutionary stage in microecological therapeutics. Their core advantage lies not only in their inherent compositional clarity and controllability at the design level, but also in their capacity to achieve a high degree of orthogonality and systems-level emergent properties.^13^ They spontaneously form complex ecological networks in which the collective functional output of the consortium substantially exceeds the simple sum of its individual members. This function is achieved through metabolic coupling mechanisms such as mutualism or cross-feeding among constituent strains.^14^

In this review, the term “SynComs” refers to rationally assembled, compositionally defined microbial consortia designed according to ecological engineering and/or synthetic biology principles. In contrast, “defined microbiota therapeutics” refers to clinically developed multi-strain products with well-defined compositions that may incorporate certain SynComs design principles while remaining conceptually distinct from canonical SynComs. To illustrate these generational distinctions, Table 1 systematically summarizes the key characteristics of the six major categories of microecological intervention strategies, ranging from conventional FMT to advanced engineered consortia across core dimensions, including definition, donor dependency, compositional definition, and regulatory pathways.^4^ ^,^ ^15-19^

**Table 1.** Comparison of key characteristics among microbiota-based therapeutic strategies across the LBP spectrum.

Therapeutic category	Definition	Strain origin	Compositional definition	Degree of engineering	Representative products	Regulatory pathways
FMT	Direct administration of donor-derived, complex gut microbial communities without strain isolation	Donor feces	Undefined and donor-dependent, with substantial heterogeneity in microbial composition, virome, metabolome, and batch-to-batch variability	None; routine physical purification, suspension, or lyophilization only	Rebyota (RBX2660) [15]	FDA-approved in the U.S. for recurrent CDI; mostly limited to clinical trials or compassionate use in other countries
Donor-derived spore-based products	Donor-derived products manufactured through physical and chemical purification and enrichment of spore-forming components, without an isolation step for individual bacterial strains	Donor feces	Partially defined, restricted primarily to spore-forming Firmicutes populations	None; physical/chemical enrichment only, without genetic engineering	SER-109/VOWST [16]	Regulated under the donor-driven innovative LBP pathway; FDA label carries a clear warning regarding the risk of pathogen transmission
Traditional probiotics and single-strain LBPs	Single or limited numbers of naturally isolated and purified strains	Pure culture isolates	Defined strain composition is relatively well characterized, although batch-to-batch variability in metabolic activity may occur	None to minimal; primarily harness the physiological properties of naturally occurring wild-type strains with favorable traits	*E. coli* Nissle 1917, L. plantarum [17]; single-strain LBPs in clinical development (e.g., ADS024) [18]	Regulated as foods or pharmaceuticals, with well-established regulatory pathways
Defined microbiota therapeutics	Multi-strain consortia composed of known bacterial species, with each strain independently isolated, purified, and taxonomically identified; typically derived through top-down selection from natural microbial communities	Purified single strains selected through niche-/function-complementarity-based screening platforms	Defined taxonomic identity and whole-genome information of all selected strains are clearly known	Low to moderate; focuses on rational combination of naturally occurring superior isolates, without genetic engineering	VE303 (8 strains) [12]; MET-2 (40 strains) [13]	Standard regulatory framework for multi-strain LBPs; currently the mainstream pathway for clinical translation
SynComs	Rationally designed microbial consortia integrating top-down ecological deconstruction and bottom-up assembly	Multiple well-characterized purified strains with defined genetic backgrounds	Defined strain identities, strain counts, and genetic backgrounds are all well characterized, enabling rational reconstruction and batch-to-batch reproducibility	Low to moderate; primarily based on rational strain selection without genetic modification	Experimental multi-strain consortia rationally designed for IBD or colorectal cancer	FDA/EMA have established LBP regulatory frameworks, but specific manufacturing and quality control guidelines for multi-strain SynComs remain to be established [4]
Engineered microbial consortia	Multi-strain consortia subjected to deep genetic modification through the introduction of synthetic biology gene circuits (e.g., biosensors, logic gates)	Genetically recombinant or artificially modified strains	Defined genetic elements and synthetic regulatory networks are incorporated	Extremely high (integrates synthetic biology tools, enabling in situ responsiveness and drug delivery capabilities)	Engineered commensal consortia with in situ anti-inflammatory or anti-tumor drug delivery functions	Subject to the dual and exceptionally stringent regulatory scrutiny of both GMO regulations and multi-strain LBP frameworks, making clinical translation particularly challenging [19]

### 2.2. Design principles

SynComs are constructed via the iterative “Design–Build–Test–Learn” cycle; the process adheres to the following core design principles:

#### 2.2.1. Modular assembly

SynComs design applies distributed metabolic logic to overcome the metabolic burden of a single engineered strain when executing complex tasks. This principle prioritizes the decomposition of complex intervention tasks, such as anti-inflammatory signal transduction, pathogen antagonism, and toxin degradation, into relatively independent fundamental functional modules. Allocation of these modules to specialized strains with complementary ecological niches enables cross-species division of labor and cooperation, thereby optimizing the overall system performance and yielding biological systems with higher-order functions.^20^ ^,^ ^21^ For example, in a typical metabolic division-of-labor design, primary degraders break down complex dietary fibers into oligosaccharides. Secondary fermenters subsequently convert these oligosaccharides into butyrate. This cascade markedly improves metabolic efficiency.^22^

#### 2.2.2. Enhancement of ecological homeostasis and robustness

The long-term efficacy of a consortium depends on its robustness in complex and fluctuating intestinal environments. Therefore, the design emphasis has shifted from random co-culture to the construction of non-random interaction networks. This is achieved by: 1) establishing metabolic coupling mechanisms, such as cross-feeding, to strengthen obligate mutualistic symbiosis among members^23^; 2) employing microencapsulation technologies or guided spatial colonization that mimic the spatial heterogeneity of natural niches to mitigate competitive exclusion^24^; and 3) integrating quorum sensing-mediated negative feedback control circuits to achieve autonomous regulation of population dynamics. This approach ensures that the system maintains niche homeostasis even under external perturbations.^22^

#### 2.2.3. Safety lock-in and ecological containment

A rigorous safety design constitutes both an ethical boundary and a technical prerequisite for the clinical translation of SynComs. In current engineering strategies, multiple “safety lock” mechanisms are constructed by engineering auxotrophic chassis strains to establish survival dependence on exogenously supplied specific nutrients or by integrating controllable lysis devices through synthetic logic gate circuits. This ensures that the engineered bacteria can respond to environmental signals and automatically trigger programmed cell death upon completion of the intended therapeutic task or escape from the target habitat. This effectively minimizes the risk of horizontal transfer of engineered genetic elements and environmental colonization.^21^

### 2.3. Design strategies

The assembly of SynComs has evolved from conventional empirical trial-and-error methods toward an engineering paradigm grounded in rational design and efficient validation. At the technical core of this paradigm lie the strategic selection of assembly methods, deployment of high-throughput screening platforms, and computationally driven precision prediction.

#### 2.3.1. Synergistic strategies of “Top-Down” and “Bottom-Up” approaches

**Figure 1.**
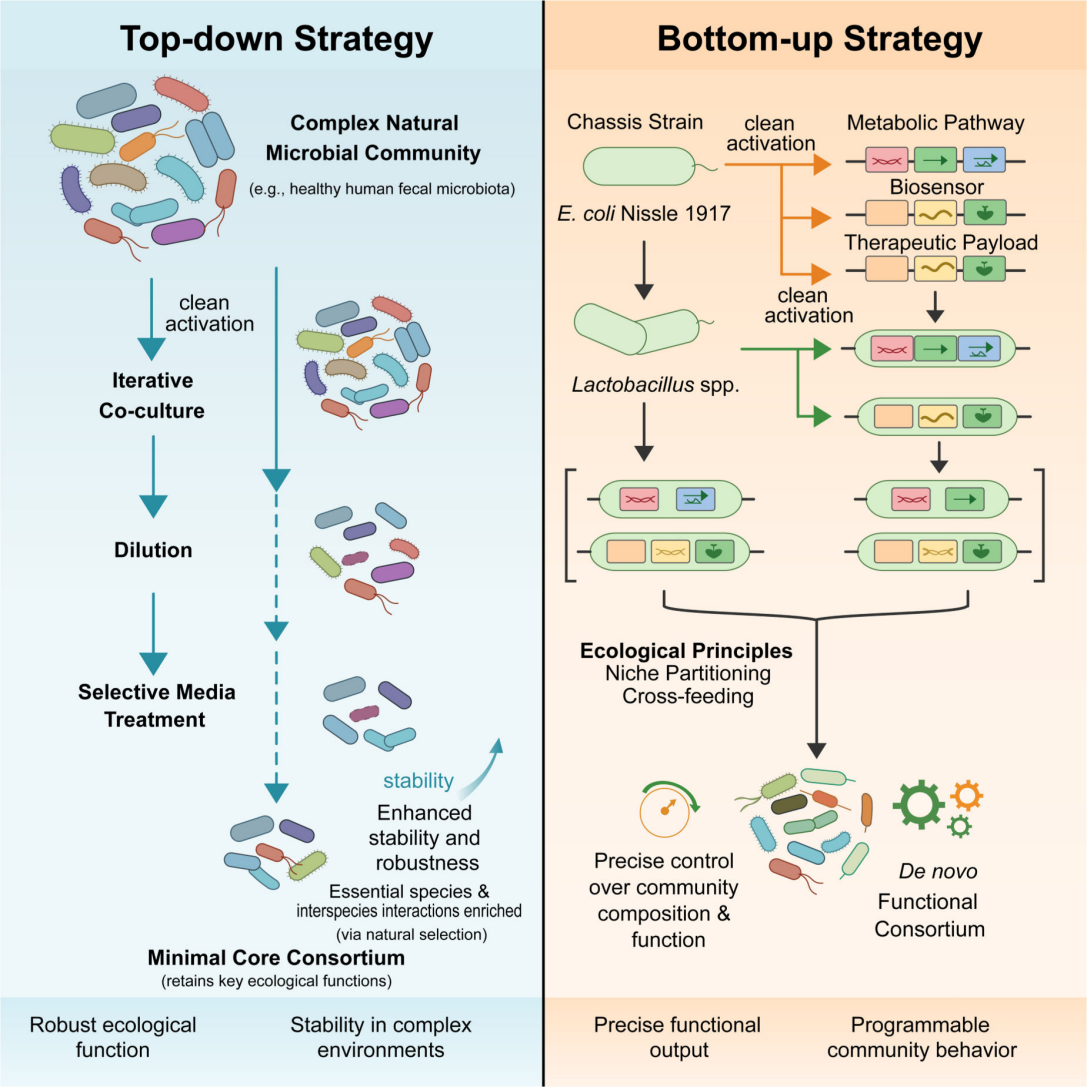
Construction strategies for synthetic microbial communities (SynComs). (Left) Top-down strategy: Complex natural communities are progressively simplified via iterative co-culture, dilution, and selective media to derive a minimal core consortium with robust ecological functions. (Right) Bottom-up strategy: Genetically tractable chassis strains are engineered with modular genetic circuits and rationally assembled based on ecological principles (e.g., niche partitioning and cross-feeding) to create *de novo* functional consortia.

The construction of SynComs primarily employs two complementary methodological approaches (Figure 1).^25-30^ The top-down deconstruction strategy progressively simplifies complex natural microbial ecosystems (e.g., human fecal microbiota) through dilution-based culturing, differential screening, or evolution under selective pressure to identify the “minimal core consortia” that sustain essential ecological functions.^31^ This approach is uniquely advantageous, as it dissects the niche contributions of keystone populations and elucidates microbiota–host interaction phenotypes in the gut.^32^ In contrast, the bottom-up assembly paradigm begins with chassis strains of well-characterized genetic backgrounds, such as *Escherichia* coli Nissle 1917 and Lacticaseibacillus species. These strains are equipped with specific sensory or therapeutic functions via genetic engineering, resulting in the construction of *de novo* synergistic consortia based on the theory of niche complementarity.^20^ For example, ecological principles such as cross-feeding enable the de novo assembly of rationally designed microbial consortia in which primary degraders are paired with butyrate-producing anaerobes. This ensures stable engraftment within the inflamed gut ecosystem and facilitates the targeted repair of the intestinal mucosal barrier.^33^ ^,^ ^34^

In summary, the “top-down” and “bottom-up” approaches exhibit conceptually opposing yet scientifically complementary properties in terms of their reconstitution logic. To overcome the translational bottlenecks inherent to either paradigm in isolation, these approaches are increasingly integrated in modern microecological medicine. Multi-omics data from the “top-down” perspective are leveraged to parse native network topology and guide the identification of core functional strain targets, followed by rational assembly through computational systems biology from the “bottom-up” perspective.^35^ This hybrid paradigm reconciles ecological robustness with mechanistic clarity and is emerging as the core methodology for the development of LBPs. To provide a more systematic illustration of these methodological distinctions, Table 2 presents a clear comparison of the two principal construction strategies across key translational dimensions, including their underlying logic, core advantages, primary disadvantages, applicable disease scenarios, and translational limitations.

**Table 2.** Comparative analysis of top-down and bottom-up SynCom construction strategies.

Dimensions	Core logic	Advantages	Disadvantages	Scenarios	Limitations
Top-down Approach	Starting from complex natural communities (e.g., fecal samples) and applying environmental pressures or in vitro selection as a “subtractive” approach to extract core functional networks [25]	Maintains intact natural co-evolved networks with robust engraftment and high adaptability	Mechanistically less resolved (“black box”); prone to functional redundancy and potential antimicrobial resistance genes	CDI: core microbiome screening [27]; UC: phage-assisted community evolution [28]	Co-fermentation kinetics of multiple strains are inherently complex; maintaining batch-to-batch consistency at scale is exceptionally challenging
Bottom-up Approach	Chassis strains with defined genetic/metabolic backgrounds; computational modeling combined with combinatorial assays for de novo assembly [26]	Fully defined (“white-box”); high target specificity and engineering potential	Oversimplified networks; vulnerable to host immunity and resident microbiota clearance in vivo	IBD: butyrate-producing SynComs [29]; CRC immunomodulatory consortia [30]	High computational costs for screening; no microphysiological systems mimicking host in vivo environment

#### 2.3.2. High-throughput validation and automated screening

High‑throughput screening technologies enable the identification of optimal functional consortia from a vast pool of candidate strain combinations.^36^ By leveraging droplet-based microfluidics, automated robotic workstations, and microphysiological systems, researchers can characterize the spatiotemporal dynamics and functional outputs of thousands of microbial communities in parallel.^37^ This not only markedly accelerates the development cycle but also generates high-throughput phenotypic data that are essential for elucidating complex nonlinear interactions among consortium members.

#### 2.3.3. Computational modeling and AI-driven design optimization

The convergence of computational systems biology and data science is expected to usher SynComs into a new era of “prediction-driven construction.” At the metabolic modeling level, genome-scale metabolic models employing flux balance analysis enable the quantitative simulation of metabolite exchange networks within a consortium. These models predict growth dynamics, stability, and the spatiotemporal distribution of products, thereby guiding the initial screening of strain combinations before wet-lab experiments.^38^ In artificial intelligence (AI) and machine learning, algorithms such as random forests and neural networks are harnessed to mine multi-omics data and learn from limited experimental datasets to forecast the evolutionary trajectories and functional potential of large-scale strain combinations.^39^ ^,^ ^40^ Generative AI is also being explored for strain function prediction. However, its practical utility in SynComs design warrants further validation.

This “computation–experimentation” coupled paradigm wherein model-based predictions guide and are validated by automated experimental feedback in a closed loop has become a critical foundation for the construction of complex, robust SynComs with significant potential for clinical translation.

## 3. Mechanisms through which SynComs combat intestinal pathogen infections

**Figure 2.**
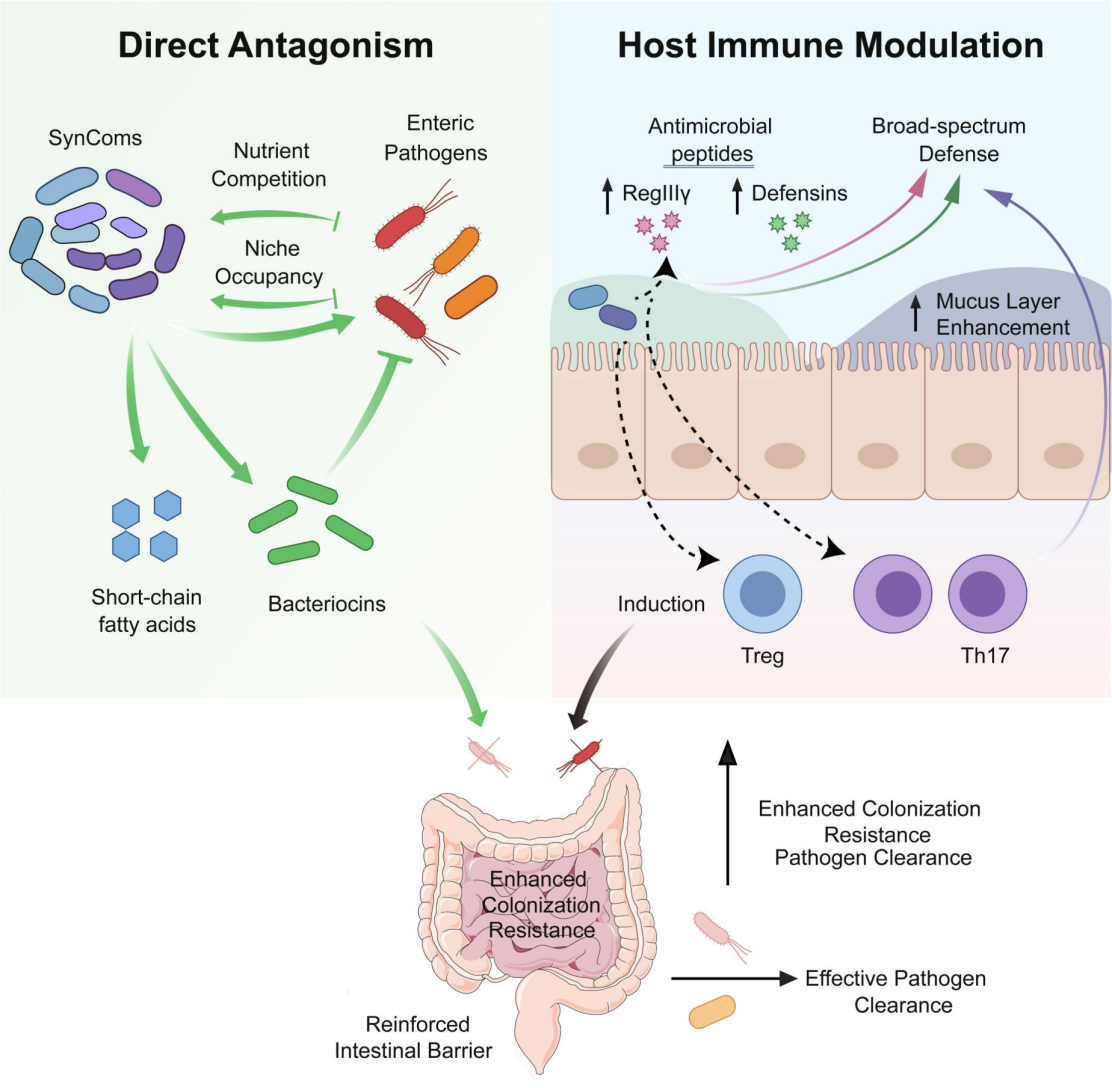
Dual protective mechanisms of SynComs against enteric pathogens. SynComs establish colonization resistance through (left) direct antagonism, including nutrient competition, niche occupancy, and the secretion of antimicrobial metabolites (e.g., SCFAs and bacteriocins). Concurrently, SynComs trigger (right) host immune modulation by stimulating innate defenses (e.g., RegIII-*γ* and mucus secretion) and shaping adaptive immunity (e.g., Treg and Th17 induction). The synergy of these pathways enhances barrier integrity and ensures effective pathogen clearance.

SynComs establish multilayered biological barriers through synergistic integration of direct antagonism and host immune modulation. Figure 2 schematically depicts comprehensive defense pathways encompassing mechanisms such as physical barrier reinforcement, biochemical inhibition, and immune system education.

### 3.1. Universal mechanisms of direct antagonism and colonization resistance

Direct antagonism constitutes the fundamental mechanism by which the indigenous gut microbiota mediates colonization resistance. Through rational design, SynComs not only mimic and reinforce this innate defense, but also endow it with a high degree of precision. SynComs member strains competitively deplete key metabolic substrates (e.g., specific sulfur-containing amino acids or monosaccharides) and occupy physical mucosal niches through ecological exclusion via resource and spatial competition, thereby establishing physical and nutritional barriers against pathogens such as *Salmonella Typhimurium* and *C. difficile*.^40^ Regarding targeted metabolic inhibition, SCFAs, secondary bile acids, and bacteriocins (e.g., nisin) produced by member strains establish a biochemical barrier through acidification of the microenvironment or direct disruption of pathogen cell membrane integrity. Secondary bile acids (e.g., deoxycholic acid) do not effectively trigger *C. difficile* spore germination; instead, they competitively bind to spore germination receptors, thereby antagonizing germination signals mediated by primary bile acids (e.g., taurocholate). This constitutes a critical chemical barrier through which the gut microbiota prevents *C. difficile* colonization.^41^ The core logic underlying this interspecies antagonism lies in competition within metabolic networks and quorum sensing interference. Regardless of the phylogenetic relatedness of their members, SynComs can reshape resource flow via overlapping metabolic pathways or disrupt the coordinated expression of pathogenic virulence factors via the secretion of quorum-quenching enzymes. This multimodal intervention, operating across the physical, chemical, and signaling dimensions, provides a broadly applicable theoretical foundation for the application of SynComs as an alternative to antibiotics.^5^ ^,^ ^6^

### 3.2. Indirect host immune defense and systemic co-development

Beyond direct antagonism, SynComs function as “educators” that shape the host immune landscape, with mechanistic insights evolving from descriptive phenotypes toward the elucidation of specific metabolite–receptor–signaling pathways. SCFAs produced by SynComs member strains, particularly butyrate and secondary bile acid derivatives (e.g., *ω*-muricholic acid and isoallolithocholic acid) are pivotal messengers linking microbial metabolism with host immunity. Butyrate upregulates the expression of antimicrobial peptides (e.g., RegIII *γ* and *β*-defensin) in colonic epithelial cells by inhibiting HDAC activity. It also directly acts on dendritic cells (DCs) via the pregnane X or vitamin D receptors, thereby driving DC differentiation toward a tolerogenic phenotype that promotes the polarization of naïve T cells into Treg cells and concurrently suppresses excessive Th17 cell activation.^42^ Secondary bile acids modulate inflammasome activity in the gut lamina propria macrophages through FXR and TGR5, thereby maintaining immune homeostasis and jointly regulating the Treg/Th17 balance to establish long-term intestinal immune tolerance.^7^ ^,^ ^31^

Interventional potential during the “developmental window”: The immunomodulatory effects of SynComs hold considerable translational value in young hosts. The core mechanism involves the establishment of a homeostatic equilibrium between immune tolerance and defense within the microbe–host immune co-development” window. During the critical phase of immune maturation, early colonization with rationally designed SynComs recapitulates the evolutionary logic of natural pioneer species, effectively “training” the immature immune system to establish a healthy immune tone, thereby preventing the subsequent onset of allergic diseases or chronic inflammatory conditions.^43^

### 3.3. Synergistic effects of the dual mechanisms

Direct antagonism and immune modulation are not merely additives. They constitute an integrated synergistic system that couples immediate efficacy with long-term protection. SynComs leverage niche occupation to establish a critical temporal window for defense, whereas sustained immune stimulation enhances the “depth” of host defense.^6^ ^,^ ^44^ Theoretically, this rationally designed synergistic mechanism aligns with and transcends the evolutionary strategies of the natural microbiota. Synergy within natural consortia is frequently characterized by substantial stochasticity. In contrast, SynComs emphasize “rational reconstitution;” bypassing random screening to directly couple effective ecological competition with targeted immune training in a modular fashion. This design not only preserves the biological role of natural communities in maintaining homeostasis but also employs engineering approaches to enhance response rapidity and broad-spectrum defense efficacy. Thus, it provides a promising framework for addressing the formidable challenge of antimicrobial resistance.

## 4. Advances in SynComs applications

### 4.1. CDI: from donor-derived to compositionally defined microbiota therapeutics

CDI is the most advanced application for the clinical translation of microbiota-based therapeutics. In this field, intervention strategies are steadily evolving from empirical microbiota transplantation to compositionally defined microbiota therapeutics with increasing levels of rational design. Recently approved donor-derived microbiota therapeutics, including the rectally administered RBX2660 (Rebyota™) and the oral fecal spore-based formulation SER-109 (Vowst™), have achieved important breakthroughs in preventing recurrent CDI. However, these products are donor-dependent and compositionally undefined formulations derived from human fecal material without the isolation of individual strains. Consequently, their official U.S. Food and Drug Administration (FDA) labeling provides a warning regarding the potential risk of pathogen transmission.

To overcome the translational bottlenecks of donor dependency and undefined composition, the field is advancing toward emerging defined microbiota therapeutics. Among these, VE303 is one of the most advanced clinical products. In the Phase II CONSORTIUM study, high-dose VE303 was well-tolerated and reduced the incidence of recurrent CDI by more than 80% compared to the placebo. Multi-omics dynamic modeling demonstrated that VE303 markedly accelerated the early restoration of the gut microbiome following antibiotic treatment, selectively enhanced SCFA and secondary bile acid production, and markedly upregulated the expression of bile salt hydrolase genes, thereby establishing robust colonization resistance. VE303 has now entered the global Phase III RESTORATiVE303 registration trial (NCT06237452).^11^ Although it is compositionally defined and rationally assembled based on ecological principles, VE303 is more appropriately regarded as a defined microbiota therapeutic that incorporates key design principles, such as functional niche complementarity, rather than as a fully engineered canonical SynComs. MET-2, another compositionally defined microbiota therapeutic comprising 40 purified bacterial strains, demonstrated a microbiota restoration efficacy comparable to that of FMT in a Phase I trial. This further highlights the feasibility of donor-independent, defined microbiota-based therapies.^12^

Collectively, these developments illustrate the ongoing transition from donor-dependent microbiota products to donor-independent, compositionally defined microbiota therapeutics. Table 3 illustrates the evolutionary trajectory of microbiota-based therapeutics from complex donor-derived microbial mixtures to compositionally defined microbiota therapeutics and compares their translational characteristics. Specifically, Table 3 summarizes the microbial composition, derivation strategy, clinical indication, development stage, key efficacy data, proposed mechanisms of action, and current limitations of conventional FMT, donor-derived microbiota therapeutics (Rebyota and SER-109), and representative defined microbiota therapeutics (VE303 and MET-2).^11^ ^,^ ^16^ ^,^ ^45^ ^,^ ^46^

**Table 3.** Comparative summary of representative CDI-targeted products: from donor-derived to compositionally defined microbiota therapeutics.

Product	Microbial composition	Derivation strategy	Indication	Clinical phase	Key efficacy data	Mechanism	Limitations
Rebyota™ (RBX2660) [45]	Broad-spectrum fecal microbiota (spore and non-spore forming)	Donor-derived, undefined composition	Prevention of rCDI	FDA approved (2022)	Superior to placebo in reducing rCDI	Restoration of diverse gut microbiota	Donor dependency; batch-to-batch variability; pathogen transmission risk
Vowst™ (SER-109) [16]	Spores from approximately 50 Firmicutes species	Donor-derived, ethanol-purified spores; undefined composition (without single-strain isolation step)	Prevention of rCDI	FDA approved (2023)	8-week rCDI: 9.5% (95% CI 6.6–13.0); 24-week: 15.2%	Secondary bile acid-mediated inhibition of C. difficile spore germination	Donor-sourced starting material; limited capacity for mechanistic reconstruction
VE303 [11]	8 clonally purified bacterial strains	Compositionally defined consortium; rationally selected via ecological niche complementation; no donor dependency	Prevention of rCDI	Phase III (RESTORATiVE303)	>80% reduction in the odds of rCDI vs. placebo (Phase II)	Multi-modal: SCFA/secondary bile acid restoration; reduced epithelial stress and inflammation	Phase III data pending; defined but not genetically engineered
MET-2 [46]	40 selected bacterial strains	Compositionally defined consortium; derived from a single donor but isolated and grown independently	rCDI; also-under investigation for other indications	Phase I complete	Safe and well tolerated; microbiome effects comparable to those observed after FMT	Microbiome restoration and anaerobe repletion	Phase I only; controlled studies required to validate efficacy

### 4.2. Inflammatory bowel disease: from immunomodulation to barrier repair

IBD, which primarily encompasses Crohn’s disease and ulcerative colitis, is another core indication for SynComs research. Building on the immune regulatory mechanisms detailed in Section 3.2, SynComs ameliorates IBD via the following pathways:

(1) Restoration of the Treg/Th17 balance.

Butyrate-producing bacteria (e.g., *Faecalibacterium prausnitzii* and *Roseburia intestinalis*) promote Treg differentiation via HDAC inhibition, thereby suppressing Th17-mediated intestinal inflammation.^47^ ^,^ ^48^ Kurt^49^ constructed a nine-strain synthetic consortium whose efficacy in correcting dysbiosis was comparable to that of FMT in a DSS-induced acute colitis mouse model. In contrast, an unstructured mixture of these strains failed to produce equivalent effects, underscoring the importance of the functional division-of-labor design.

(2) Enhancement of intestinal barrier function.

SynCom-derived SCFAs promote IL-22 production by CD4⁺ T and innate lymphoid cells through signaling via the G-protein-coupled receptor and HDAC inhibition. IL-22, in turn, upregulates the expression of tight junction proteins (e.g., occludin and claudin-5), reduces intestinal permeability, and limits bacterial translocation,^50^ thereby preventing inflammation in the gut.

(3) Clearance of adherent‑invasive *E. coli* (AIEC).

Engineered SynComs can secrete bacteriocins or competitively occupy the mucus niche to decrease AIEC colonization in the ileal mucosa. Multiple LBP candidate strains have been shown to effectively inhibit the adhesion to and invasion of intestinal epithelial cells by AIEC. For instance, pre-inoculation with LBPs reduced AIEC invasion by up to 97% in an intestinal epithelial cell adhesion model.^51^

Status of clinical translation. Despite robust preclinical evidence, Phase II/III clinical trials of SynComs for IBD have not yet been reported. Existing LBPs such as ADS024 (a single-strain product) have shown anti-inflammatory and barrier-protective effects in DSS-induced colitis mouse models.^18^ However, human data on multi-strain synergistic SynComs in IBD remain absent, representing a critical gap in the field that urgently needs to be addressed.

### 4.3. CRC: metabolic prevention and immunotherapy sensitization

#### 4.3.1. Metabolic prevention

Gut dysbiosis is closely associated with the development and progression of CRC. SynComs exert protective effects by introducing or enriching butyrate-producing strains such as *Eubacterium rectale* and *F. prausnitzii*. Butyrate serves as the primary energy source for colonic epithelial cells and suppresses inflammation and tumor cell proliferation through HDAC inhibition.^52^ Furthermore, modulation of secondary bile acid metabolism may reduce CRC risk. SynCom members such as *Clostridium scindens* can convert primary bile acids into non-carcinogenic derivatives, thereby diminishing the accumulation of tumor-promoting metabolites such as deoxycholic acid.^53^

#### 4.3.2. Sensitization to immune checkpoint inhibitors

The composition of the gut microbiota is closely associated with the response rate to anti-PD-1/PD-L1 therapy. SynComs can potentiate the efficacy of immune checkpoint inhibitors (ICIs) by reshaping the gut–immune axis. For instance, a synthetic community isolated from ICI responders (e.g., RCom) can increase CD8⁺ T cell infiltration into the tumor microenvironment and attenuate the activity of myeloid-derived suppressor cells, thereby overcoming resistance attributable to inter-individual gut microbiota heterogeneity.^54^ Although this evidence has been primarily derived from extraintestinal tumors such as melanoma, the principles governing microbiota-mediated modulation of ICI sensitivity are applicable to CRC. Moreover, studies on hepatocellular carcinoma have demonstrated that *Phocaeicola vulgatus* is markedly enriched in the gut of patients who are not responsive to PD-1 inhibitors, where it impairs CD8⁺ T cell function by suppressing the production of indole-3-acetic acid (IAA).^55^ This suggests that targeted depletion of such negative regulatory bacteria via SynComs or supplementation with IAA may represent a promising adjuvant strategy for reversing ICI resistance.

## 5. Translational bottlenecks and strategies for SynComs

Current preclinical studies and limited early-phase clinical evidence suggest the potential of SynComs. However, their systematic translation from the bench to the bedside presents multiple challenges (Table 4). Nevertheless, these bottlenecks should not be interpreted as negating the inherent feasibility of SynComs; rather, they represent engineering-intensive hurdles that any LBPs must overcome during the transition from proof-of-concept to standardized manufacturing. The following section examines the most critical limiting factors—colonization resistance, chemistry, manufacturing scalability (CMC), biosafety assurance, and regulatory uncertainty—and proposes feasible strategies for addressing each challenge.

**Table 4.** Translational bottlenecks and potential solutions for SynCom-based therapeutics.

Dimensions	Key bottlenecks	Potential strategies	Section
Colonization Resistance	Preferential utilization of nutritional substrates, physical occupation of ecological niches, and secretion of antimicrobial compounds by the resident microbiota [6]	(i) selecting native dominant commensals (e.g., *F. prausnitzii*, *Bacteroides*) as chassis strains [59]; (ii) implementing personalized/stratified rationally designed assembly based on the patient’s baseline microbiota [56]; (iii) employing strategic dietary or antibiotic pretreatment to create a cleared niche [60]; and (iv) integrating anti-predation traits such as biofilm formation—to enhance persistence under natural biological pressures [61]	Section 5.1
CMC & Scale-up	Inter-strain variations in growth rates, nutritional preferences, and shear stress sensitivity drive community structure drift during large-scale fermentation; differential stress tolerance during lyophilization may result in compositional imbalance in strict stoichiometric ratios [51,62]	(i) adopting stepwise fermentation with independent strain cultivation followed by rational proportional blending; (ii) implementing real-time process analytical technology (PAT) and multi-omic dynamic monitoring; and (iii) developing advanced independent microencapsulation technologies	Section 5.2
Biosafety & HGT Risk	Frequent horizontal gene transfer (HGT) within the dense gut "mobilome" poses a risk of transfer of engineered genetic elements into resident commensals, such as antibiotic resistance markers and synthetic gene circuits [63,64]	(i) deploying CRISPR-Cas-based sequence specific lethal “kill switches” [65]; (ii) introducing xenobiology designs that strictly depend on non-canonical amino acids (ncAAs) for survival	Section 5.3
Regulation & Evaluation	Conventional PK/PD models may not be directly applicable to self-replicating live microbial therapeutics for self-replicating “live drugs” [69]; and there is a lack of consensus on quality control standards for evaluating synergistic/antagonistic effects and batch-to-batch consistency in multi-strain consortia [67,68]	(i) establishing novel MK and MD evaluation frameworks to systematically quantify ecological washout periods; and (ii) advancing the standardized application of in vitro simulation platforms (e.g., SHIME) and microfluidic gut‑on‑a‑chip technologies [71,73]	Section 5.4

### 5.1. Colonization resistance: bridging the gap from “efficacy in germ‑free models” to “engraftment in complex hosts”

This constitutes the most fundamental biological barrier to the translation of SynComs. Colonization and functional data validated in germ-free animal models are often poorly recapitulated in conventional hosts harboring an intact, saturated microbiota. This is attributed to colonization resistance established by the indigenous microbiota through long-term co-evolution, which manifests as the preferential utilization of key nutrient substrates, physical occupation of mucosal niches, and sustained secretion of broad-spectrum antimicrobials.^44^ Current SynCom design largely relies on the paradigm of “screening for optimal functional combinations in a niche vacuum,” without incorporating the host’s resident microbiota as a core constraint.^56^ Feasible strategies for overcoming this impasse include: (i) directly selecting chassis strains from the dominant commensals of the target population, such as *F. prausnitzii*, butyrate-producing strains, and certain *Bacteroides* species, to leverage their intrinsic host adaptation. This approach harnesses the inherent host-adaptive capabilities and competitive advantages of these strains to considerably enhance colonization stability and functionality^57^; (ii) designing microbiome-guided consortia that incorporate the patient’s baseline microbiota profiles as a core design constraint, thereby enabling personalized strain combinations tailored to individual ecological niche availability. This strategy shifts the design paradigm from a “one-size-fits-all” consortium to “patient-stratified” or even “patient-personalized” synthetic consortia^55^; (iii) niche-creating ecological engineering, in which targeted antibiotic or dietary pretreatment is used to temporarily create ecological niches that facilitate SynCom engraftment, thereby effectively lowering the colonization resistance barrier before administration^58^; and (iv) integrating predator resistance traits as a design criterion, including biofilm formation and the production of anti-predator metabolites, to enhance the resilience and persistence of SynComs under natural biological pressures.^59^

### 5.2. The chemistry, manufacturing scalability bottleneck: challenges in large-scale manufacturing and stoichiometric control

CMC constitutes a critical bottleneck in the industrialization of SynComs. Inherent inter-strain variations in specific growth rates, nutritional preferences, and shear stress sensitivity render large-scale fermentation highly susceptible to community structure drift.^49^ ^,^ ^60^ Moreover, maintaining strict stoichiometric ratios of the consortium remains a formidable challenge during downstream processing, particularly lyophilization. This difficulty arises because differential resistance to dehydration and osmotic stress among community members can lead to an imbalanced viability of the key strains. Strategies such as stepwise fermentation, proportional blending of individually cultured strains, and advanced microencapsulation are being explored to address these issues. However, there is an urgent need to integrate real-time process analytical technology and multi-omics monitoring to ensure batch-to-batch consistency and compositional fidelity.

### 5.3. Biosafety and genetic biocontainment in the gut “mobilome”

Regarding biosafety, the human gastrointestinal tract functions as a dense and highly dynamic “mobilome” characterized by frequent horizontal gene transfer via conjugative plasmids, bacteriophages, and transposons. The risk of engineered genetic elements, such as antibiotic resistance markers or synthetic gene circuits, escaping into wild-type pathogens or commensals following SynCom administration has not been systematically evaluated.^61^ ^,^ ^62^ Thus, robust genetic biocontainment strategies must be implemented to safely deploy synthetic consortia. Emerging designs are moving beyond simple auxotrophy toward advanced “kill switches.” These include CRISPR-Cas-based sequence-specific lethal systems^63^ that degrade the engineered genome upon departure from the host environment. Similarly, xenobiology approaches such as dependence on non-canonical synthetic amino acids strictly restrict the survival of synthetic consortia to the target physiological microenvironment.

### 5.4. Regulation and standardization

Similar to LBPs, the development and clinical translation of SynComs face unique regulatory challenges. In human medicine, the FDA has progressively incorporated live-biotherapeutic-based products into its regulatory framework,^64^ requiring the characterization of strain identity, genetic stability, purification processes, and safety and efficacy data. Similarly, the European Medicines Agency has issued guidelines emphasizing the risk assessment of genetic modifications in engineered strains, analysis of colonization kinetics, and monitoring of long-term ecological effects. Nevertheless, significant gaps persist in the current regulations governing SynComs as multi-strain composite products. For instance, there is no clear consensus on defining synergistic effects versus potential antagonism among constituent strains,^65^ establishing batch-to-batch consistency standards, or evaluating the safety of interactions with the host microbiota.^66^

The scientific basis for this regulatory lag lies in the limited validity of conventional PK/PD models developed for traditional chemical drugs or biological macromolecules in SynComs. For “live drugs” capable of self-replication and engraftment, unidirectional pharmacokinetic parameters such as “half-life” or “maximum plasma concentration” hold no meaningful physical or biological value.^67^ This highlights the urgent need for novel evaluation frameworks—termed microbiokinetics and microbiodynamics—that leverage highly sensitive absolute quantification technologies to systematically measure the colonization kinetics, baseline activity, abundance thresholds, and metabolite secretion dynamics of SynComs in the gut, while also defining their clearance rates and ecological washout periods. Such frameworks would provide a scientific basis for clinical dosing regimens and facilitate a paradigm shift in regulatory assessment from “experience-driven” to “data-driven dynamic modeling.”^68^ Given the substantial inter-individual heterogeneity of the host microbiome, regulatory agencies and the research community should jointly promote the standardized application of *in vitro* simulation platforms (e.g., the SHIME system) and microfluidic gut-on-a-chip technologies.^69-71^ These platforms not only circumvent confounding factors associated with complex host physiology but also yield highly reproducible, mechanistically rich ecological and metabolic data. Incorporating such data as high-quality preclinical submission materials could effectively support the transition of SynComs from laboratory proof-of-concept to standardized clinical trials, ultimately steering regulatory pathways from reactive “case-by-case” approvals toward proactive “pathway-defined” frameworks.

## 6. Challenges and future perspectives

As emerging live biotherapeutics targeting the gut microbiome, SynComs, have demonstrated substantial potential both as specific interventional agents and as a platform technology. Their core concepts, design principles, and technological advances have been systematically supported by empirical evidence, and a clear consensus has emerged across interdisciplinary fields.

Nevertheless, the interactions of SynComs with the host and other microorganisms are considerably complex and their effects can be double-edged, underscoring the need for a rational design. For instance, the co-colonization of sulfate-reducing bacteria with certain synthetic consortia may exacerbate intestinal permeability and hepatic inflammation.^72^ This highlights the critical need for comprehensive elucidation of the metabolic networks and interaction dynamics of synthetic consortia during their design and deployment to avoid adverse effects. The rapid development of advanced technological platforms, including multi-omics approaches, AI, gut-on-a-chip systems, and organoids, provides a basis for addressing the aforementioned complexities and ensuring design precision.^73^ Building on these advances, future developments in SynComs should focus on the following key areas:

### 6.1. From generic chassis to tailored autochthonous strains

Current SynComs construction predominantly depends on established laboratory model strains (e.g., *E. coli* Nissle 1917 and lactic acid bacteria). These strains possess a certain degree of colonization capacity. However, their colonization efficiency and functional efficacy are often constrained across diverse host backgrounds, particularly in patients with severely disrupted microbiota. Therefore, future research should prioritize the isolation and genetic engineering of strains with intrinsic colonization advantages from healthy human gut microbiota, such as *F. prausnitzii*, butyrate-producing strains, and specific *Bacteroides* species. This “tailored chassis” strategy will address the fundamental challenges of host adaptation, thereby substantially enhancing the colonization stability and functional persistence of SynComs in targeted populations.^74^

### 6.2. Integration of diagnosis and therapy

Traditional SynComs typically operate constitutively (i.e., continuously secreting therapeutic molecules) or inducibly (requiring exogenous inducers), consequently precluding adaptive responses to disease states.^75^ Therefore, a key future direction is the engineering of intelligently responsive SynComs that can precisely recognize pathological conditions and trigger therapeutic functions such as the secretion of bacteriocins or anti-inflammatory factors upon sensing pathogen invasion or inflammatory signals. This capability is achieved by integrating sensors specific to pathogens (e.g., *Salmonella* quorum-sensing molecules or *“C. difficile” toxins*) or disease biomarker sensors (e.g., inflammatory cytokines and hypoxia signal*s*).^76^ ^,^ ^77^ This “context-dependent (or stimulus-responsive)” therapeutic paradigm not only minimizes disruption of the resident microbiota but also mitigates ecological safety risks associated with long-term colonization of engineered bacteria.

### 6.3. Synergy of SynComs with diverse biotechnology platforms

The functional boundaries of individual SynComs can be expanded through cross-disciplinary integration with other biotechnological platforms. Combining SynComs with bacteriophages establishes a “dual-targeting” system: SynComs suppress pathogens through niche competition and metabolic antagonism, whereas bacteriophages precisely eliminate established pathogens via lysis. This synergy significantly enhances anti-infective efficacy.^78^ Similarly, the integration of SynComs with nanobodies can leverage the high affinity of nanobodies and *in situ* expression capacity of SynComs, thereby enabling the sustained local delivery of therapeutic antibodies in the gut. Moreover, SynComs can be integrated with drug delivery systems, serving as “living carriers” for small-molecule drugs, nucleic acid therapeutics, or immunomodulators, thereby achieving targeted release and combination therapy.^4^ This interdisciplinary convergence holds promise for transcending the functional limitations of single SynComs and paving the way for the establishment of new therapeutic modalities.

## 7. Conclusions

SynComs stand at a critical inflection point, advancing from laboratory-scale proof-of-concept studies to early translational evaluation. Whether they ultimately become a core interventional tool for intestinal diseases will depend on the ability to adopt a truly host-centric approach that integrates synthetic biology, computational modeling, microfluidic screening, and regulatory science to construct safe, controllable, high-standard live biotherapeutic platforms. Thus, SynComs hold the potential not only to reshape the therapeutic landscape for intestinal diseases, but also to pioneer a new trajectory for microbiome medicine, moving from empirical supplementation to rational reconstitution.
